# Association of Endocrine Disrupting Chemicals With the Metabolic Syndrome Among Women in the Multiethnic Cohort Study

**DOI:** 10.1210/jendso/bvad136

**Published:** 2023-11-17

**Authors:** Ugonna Ihenacho, Cherie Guillermo, Lynne R Wilkens, Adrian A Franke, Chiuchen Tseng, Yuqing Li, Meera Sangaramoorthy, Mindy C Derouen, Christopher A Haiman, Daniel O Stram, Loïc Le Marchand, Iona Cheng, Anna H Wu

**Affiliations:** Department of Population and Public Health Sciences, Keck School of Medicine, University of Southern California, Los Angeles, CA 90089, USA; Population Sciences in the Pacific Program, University of Hawaii Cancer Center, Honolulu, HI 96813, USA; Population Sciences in the Pacific Program, University of Hawaii Cancer Center, Honolulu, HI 96813, USA; Cancer Biology Program, University of Hawaii Cancer Center, Honolulu, HI 96813, USA; Department of Population and Public Health Sciences, Keck School of Medicine, University of Southern California, Los Angeles, CA 90089, USA; Department of Epidemiology and Biostatistics, University of California San Francisco, San Francisco, CA 94158, USA; Department of Epidemiology and Biostatistics, University of California San Francisco, San Francisco, CA 94158, USA; Department of Epidemiology and Biostatistics, University of California San Francisco, San Francisco, CA 94158, USA; Department of Population and Public Health Sciences, Keck School of Medicine, University of Southern California, Los Angeles, CA 90089, USA; Center for Genetic Epidemiology, University of Southern California, Los Angeles, CA 90089, USA; Department of Population and Public Health Sciences, Keck School of Medicine, University of Southern California, Los Angeles, CA 90089, USA; Population Sciences in the Pacific Program, University of Hawaii Cancer Center, Honolulu, HI 96813, USA; Department of Epidemiology and Biostatistics, University of California San Francisco, San Francisco, CA 94158, USA; Department of Population and Public Health Sciences, Keck School of Medicine, University of Southern California, Los Angeles, CA 90089, USA

**Keywords:** metabolic syndrome, bisphenol A, triclosan, parabens, phthalate, multiethnic

## Abstract

Metabolic syndrome (MetS) is associated with a high risk of cardiovascular disease, a leading cause of death among women. MetS is a diagnosis of at least 3 of the following: high blood pressure, high fasting glucose, high triglycerides, high waist circumference, and low high-density lipoprotein cholesterol. Epidemiological studies suggest that endocrine disrupting chemical (EDC) exposure is positively associated with individual components of MetS, but evidence of an association between EDCs and MetS remains inconsistent. In a cross-sectional analysis within the Multiethnic Cohort Study, we evaluated the association between 4 classes of urinary EDCs (bisphenol A [BPA], triclosan, parabens, and phthalates) and MetS among 1728 women. Multivariable logistic regression was used to estimate odds ratios and 95% CI for the association between tertiles of each EDC and MetS adjusting for age, body mass index (BMI), racial and ethnic group, and breast cancer status. Stratified analyses by race and ethnicity and BMI were conducted. MetS was identified in 519 (30.0%) women. We did not detect statistically significant associations of MetS with BPA, triclosan, or phthalate metabolite excretion. MetS was inversely associated with total parabens (*P*_trend_ = .002). Although there were suggestive inverse associations between EDCs and MetS among Latino and African American women, and women with BMI < 30 kg/m^2^, there was no statistically significant heterogeneity in associations by race and ethnicity or BMI. These findings suggest an inverse association between parabens and MetS in larger multiethnic studies. Prospective analyses to investigate suggested differences in associations by race, ethnicity, and BMI are warranted.

Ubiquitous exposure to endocrine disrupting chemicals (EDCs) is an ongoing public health concern. EDCs are substances that alter hormone function [1]. Bisphenol A (BPA), triclosan, parabens, and phthalates are classes of EDCs commonly found in cosmetics, cleaning, personal care, and other consumer products, resulting in absorbed, ingested, or inhaled exposure to these compounds [2, 3]. Persistent exposure to EDCs may increase the risk of the individual components of metabolic syndrome (MetS) [4-11]. MetS is characterized by having 3 or more of the following conditions: high blood pressure, high fasting glucose, high triglycerides, high waist circumference, and low high-density lipoprotein (HDL) cholesterol levels [12]. It is estimated that MetS affects about 35% of adults in the United States with a high prevalence among adults ≥60 years of age and an increasing prevalence among women, Latino, and Asian individuals [13].

In 2015, the Parma consensus statement on metabolic disruptors acknowledged the growing incidence of metabolic diseases globally, and also highlighted that the increase cannot be accounted for by classical genetic factors alone but is likely due to environmental factors as well, and research is needed to understand associations and mechanisms of EDC exposure with MetS [14]. Prior studies of EDC exposures and MetS have been inconclusive [9, 15-22], but differences in associations by race and ethnicity [18], sex [18, 19], and age [16, 19] have been reported. Because of the high and growing burden of MetS among older women, we evaluated associations of BPA, triclosan, paraben, and phthalate exposure with MetS among an older and racially and ethnically diverse population of women participating in the Multiethnic Cohort Study (MEC).

## Materials and Methods

### Study Population

The MEC is a prospective cohort study that has been described previously [23]. Briefly, men and women aged 45-75 years from 5 racial and ethnic groups (African American, Japanese American, Latino, Native Hawaiian, and non-Hispanic White) and living in Hawai‘i and California were enrolled from 1993 through 1996. Baseline questionnaires captured demographics, diet, lifestyle, and anthropometric measures. Women were asked about their menstrual history, reproductive history, and hormone therapy use. In 2001-2006, a prospective biorepository was established among 67 594 participants who provided blood and urine specimens [24, 25]. Updated information on weight, hormone therapy use, and medications was recorded at sample collection. This cross-sectional analysis leveraged a nested case–control study population assembled for a prior breast cancer study [24, 25] that included 1728 women with blood and urine biospecimens. Blood samples were processed within 4 hours of collection and frozen at −80 °C [26]. Fasting blood samples (≥8 hours since last meal) were obtained for 95% of the biorepository participants. Urine samples were either overnight (12-hour collection) or first morning urine sample collections. Institutional Review Boards at the University of Hawai‘i Cancer Center and the University of Southern California approved the study protocol and all participants signed the study's informed consent form.

### Metabolic Syndrome Definition

An individual was classified as having MetS if they reported at least 3 of the following conditions: (1) high waist circumference, >35 inches; (2) high triglycerides, ≥150 mg/dL or medication for high triglycerides; (3) low HDL cholesterol, <50 mg/dL or medication for low HDL cholesterol; (4) high blood glucose, ≥100 mg/dL or medication for high blood glucose; and (5) high blood pressure, ≥130/85 mmHg or medication for high blood pressure. Waist circumference measures were self-reported on a follow-up questionnaire (2003-2008). History of high blood pressure or hypertension medication use was based on self-report before blood draw. Triglyceride, HDL cholesterol, and glucose levels were assessed from blood samples.

### Biomarker Analysis

All serum and urine measurements were conducted at the University of Hawai‘i Cancer Center Analytical Biochemistry Shared Resource under the supervision of Dr. Adrian Franke. Blood samples were analyzed for triglycerides, HDL cholesterol, and glucose using a Cobas MiraPlus chemistry analyzer (Roche, Indianapolis, IN) [27, 28].

BPA, triclosan, 5 parabens (methylparaben, ethylparaben, propylparaben, butylparaben, benzylparaben), phthalic acid, and 10 phthalate metabolites (mono-methyl phthalate [MMP], mono-ethyl phthalate [MEP], mono-n-butyl phthalate [MBP], mono-isobutyl phthalate [MiBP], mono-benzyl phthalate [MBzP], mono 2-carboxy-hexyl phthalate [MCHP], mono-2-ethylhexyl-phthalate [MEHP], mono(2-ethyl-5-hydroxyhexyl) phthalate [MEHHP], mono(2-ethyl-5-oxohexyl) phthalate [MEOHP], mono(2-ethyl-5-carboxypentyl) phthalate [MECPP]) were measured. The analytes were measured using sensitive isotope-dilution orbitrap-based high-resolution accurate mass liquid chromatography mass spectrometry [29, 30]. Laboratory personnel were blinded to MetS status. Blind replicate samples of pooled urine (5%) were included for quality control measures and coefficients of variation were calculated. The % coefficients of variation (SD/mean concentration × 100) within batch was 21.9% for BPA, 20.6% for triclosan, 21.5% for the parabens, and 23.0% for phthalates. For all urine assays, about 0.5 mL of urine was used and creatinine excretion was measured using a Roche-Cobas MiraPlus clinical chemistry auto analyzer (Roche Diagnostics, Indianapolis, IN) with a kit from Randox Laboratories (Crumlin, UK) based on the Jaffe reaction. Analytes were adjusted for urinary creatinine excretion by dividing the analyte concentration by the creatinine concentration to account for differences in urine volume. The units for analyte excretion are given in creatinine-adjusted nanograms/milligrams (ng/mg creatinine). Analytes below the lower limit of detection (LLOD) were assigned a value half of the LLOD for the analysis. The percent of samples with values below the LLOD was low for BPA (2%), triclosan (3%), methylparaben (2%), propylparaben (1%), and 8 of the 10 phthalate metabolites and phthalic acid (<8% for each), intermediate for ethylparaben (12%) and MCHP (11%), and high for MMP (20%), butylparaben (29%), and benzylparaben (82%).

### Statistical Analysis

The distributions of sociodemographic and clinical characteristics of participants with MetS status were reported as frequencies and percentages or means and SDs with statistical comparisons by chi-square tests for categorical variables and independent sample t-tests for continuous variables. Multivariable logistic regression was used to estimate the odds ratios (ORs) and 95% CIs for associations between EDCs and MetS, overall and by racial and ethnic group. EDC levels were modeled as tertiles based on the distribution among controls in the nested case–control study population [24, 25]. We examined MetS risk in relation to the following summary paraben and phthalate metabolite variables: the sum of methyl-, ethyl-, and propylparaben (ΣMEP parabens); total parabens; MEHP%; the ratios of MEHP to secondary di-2-ethylhexyl phthalate (DEHP) metabolites: MEHHP, MEOHP, MECPP; sum of all major DEHP metabolites (ΣDEHP: MEHP, MEHHP, MEOHP, MECPP); low molecular weight phthalates (ΣLMWP: MMP, MEP, MBP, MiBP); high molecular weight phthalates (ΣHMWP: MBzP, MCHP, ΣDEHP); and total phthalate (ΣLMHMPA: sum of all 10 phthalate metabolites and phthalic acid). We found no evidence of a nonlinear relationship on the log odds scale between each EDC and MetS using fractional polynomials (data not shown). Models were adjusted for breast cancer status and age (categorical; <65 years, ≥65-74 years, ≥75 years), which were matching criteria for sample selection in the nested case–control study, as well as body mass index (BMI) at biospecimen collection (<25 kg/m^2^, normal/underweight; ≥25-30 kg/m^2^, overweight; and ≥30 kg/m^2^, obese) as a potential confounder of the association. Analyses among the total population were additionally adjusted for racial and ethnic group. Log-transformed continuous EDC measures were used to evaluate dose–response relationships with a test for trend by the Wald test. We evaluated heterogeneity in the associations by racial and ethnic group and BMI using the Wald test of the cross-product interaction terms. All hypotheses tested were 2-sided. To account for multiple hypothesis testing, we applied a Bonferroni correction for the number of individual EDCs measured (n = 18) and set a statistical significance threshold at *P* < .003. Analyses were conducted using Stata version 15.1 (StataCorp LLC, College Station, TX).

## Results

Thirty percent (n = 519) of women had MetS (Table 1). MetS status did not differ by age or breast cancer status (*P* > .05 for both) but differed significantly by race and ethnicity (*P* < .001). MetS was almost twice as high among Latino (42.8%), Native Hawaiian (41.8%) and African American (40.0%) women compared with Japanese American (26.2%) and non-Hispanic White women (22.2%).

**Table 1.** Summary of demographic and clinical characteristics of the population, overall and by metabolic syndrome status among women in the multiethnic cohort study

	Total population	Metabolic syndrome		*P* value
		No	Yes	
N (%)*^a^*	1728 (100)	1209 (70.0)	519 (30.0)	
Age at blood draw, years (mean ± SD)	66.3 ± 7.9	66.3 ± 8.0	66.3 ± 7.6	.99
Age at blood draw, years				.78
<65	816 (47.2)	570 (47.1)	246 (47.4)	
65-74	640 (37.0)	444 (36.7)	196 (37.8)	
≥75	272 (15.7)	195 (16.1)	77 (14.8)	
Invasive breast cancer status at urine collection				.58
Control	933 (54.0)	661 (54.7)	272 (52.4)	
Case	670 (38.8)	466 (38.5)	204 (39.3)	
Missing	125 (7.2)	82 (6.8)	43 (8.3)	
Race and ethnicity				<.001
African American	90 (5.2)	54 (4.5)	36 (6.9)	
Japanese American	828 (47.9)	611 (50.5)	217 (41.8)	
Latino	145 (8.4)	83 (6.9)	62 (11.9)	
Native Hawaiian	287 (16.6)	167 (13.8)	120 (23.1)	
Non-Hispanic White	378 (21.9)	294 (24.3)	84 (16.2)	
Menopausal status at urine collection				.02
Premenopausal	314 (18.2)	237 (19.6)	77 (14.8)	
Postmenopausal	1407 (81.4)	968 (80.1)	439 (84.6)	
Missing	7 (0.4)	4 (0.3)	3 (0.6)	
Parity at baseline				<.001
Nulliparous	212 (12.3)	159 (13.2)	53 (10.2)	
1 child	172 (10.0)	131 (10.8)	41 (7.9)	
2–3 children	919 (53.2)	654 (54.1)	265 (51.1)	
≥4 children	417 (24.1)	262 (21.7)	155 (29.9)	
Missing	8 (0.5)	3 (0.2)	5 (1.0)	
Hormone replacement therapy use at urine collection				.02
Never estrogen use	689 (39.9)	466 (38.5)	223 (43.0)	
Past estrogen use	583 (33.7)	417 (34.5)	166 (32.0)	
Current estrogen use alone	306 (17.7)	220 (18.2)	86 (16.6)	
Current estrogen + progesterone use	136 (7.9)	101 (8.4)	35 (6.7)	
Missing	14 (0.8)	5 (0.4)	9 (1.7)	
Smoking status at baseline				.06
Never	1015 (58.7)	733 (60.6)	282 (54.3)	
Former	500 (28.9)	339 (28.0)	161 (31.0)	
Current	198 (11.5)	129 (10.7)	69 (13.3)	
Missing	15 (0.9)	8 (0.7)	7 (1.3)	
Education at baseline				<.001
≤High school graduate	540 (31.3)	341 (28.2)	199 (38.3)	
Some college	558 (32.3)	384 (31.8)	174 (33.5)	
College graduate	315 (18.2)	243 (20.1)	72 (13.9)	
Graduate/professional school	303 (17.5)	235 (19.4)	68 (13.1)	
Missing	12 (0.7)	6 (0.5)	6 (1.2)	
Body mass index at urine collection, kg/m^2^				<.001
<25	788 (45.6)	691 (57.2)	97 (18.7)	
25-<30	572 (33.1)	365 (30.2)	207 (39.9)	
≥30	368 (21.3)	153 (12.7)	215 (41.4)	
High blood pressure before urine collection				<.001
No	804 (46.5)	741 (61.3)	63 (12.1)	
Yes	924 (53.5)	468 (38.7)	456 (87.9)	
Waist circumference before urine collection, inches				<.001
0-≤35	859 (49.7)	768 (63.5)	91 (17.5)	
>35	657 (38.0)	274 (22.7)	383 (73.8)	
Missing	212 (12.3)	167 (13.8)	45 (8.7)	
Triglycerides at urine collection, mg/dL				<.001
0-<150	1245 (72.0)	990 (81.9)	255 (49.1)	
≥150	359 (20.8)	108 (8.9)	251 (48.4)	
Missing	124 (7.2)	111 (9.2)	13 (2.5)	
High-density lipoprotein cholesterol at urine collection, mg/dL				<.001
≥50	673 (38.9)	620 (51.3)	53 (10.2)	
0-<50	929 (53.8)	476 (39.4)	453 (87.3)	
Missing	126 (7.3)	113 (9.3)	13 (2.5)	
Glucose at urine collection, mg/dL				<.001
0-<100	1378 (79.7)	1086 (89.8)	292 (56.3)	
≥100	300 (17.4)	77 (6.4)	223 (43.0)	
Missing	50 (2.9)	46 (3.8)	4 (0.8)	

^*a*^All values are frequencies and column percentages unless otherwise noted. *P* values are estimated by chi-square tests for categorical variables and by independent sample t-tests for continuous variables.

We did not observe associations for BPA or triclosan with MetS. ΣMEP and total paraben were inversely associated with MetS (*P*_trend_ = .002 for each, Table 2). In analyses by race and ethnicity, we also observed these inverse associations among Latino women (*P*_trend_ = .01 for each), but they were not statistically significant and, overall, there was no heterogeneity in the associations by race and ethnicity (*P*_heterogeneity_ = .79 and .77, respectively).

**Table 2.** Multivariable-adjusted ORs and 95% CIs for the association of urine BPA, triclosan, paraben, and phthalate metabolite measures with metabolic syndrome, overall and by race and ethnicity among women in the multiethnic cohort study

	Total Population n = 1728		African American n = 90		Japanese American n = 828		Latino n = 145		Native Hawaiian n = 287		Non-Hispanic White n = 378		*P*_het_
	MetS/ No MetS	OR (95% CI)	MetS/ No MetS	OR (95% CI)	MetS/ No MetS	OR (95% CI)	MetS/ No MetS	OR (95% CI)	MetS/ No MetS	OR (95% CI)	MetS/ No MetS	OR (95% CI)	
Bisphenol A													.35
≤0.84	200/426	1.00	18/21	1.00	78/225	1.00	29/48	1.00	47/48	1.00	28/84	1.00	
>0.84-≤1.76	158/388	0.93 (0.71-1.23)	7/17	0.51 (0.15-1.75)	69/193	1.05 (0.70-1.58)	14/20	1.18 (0.49-2.86)	37/67	0.60 (0.32-1.12)	31/91	1.05 (0.55-2.04)	
>1.76	160/392	0.96 (0.73-1.26)	11/15	0.97 (0.29-3.24)	70/193	1.14 (0.76-1.72)	18/15	1.79 (0.74-4.35)	36/51	0.71 (0.38-1.35)	25/118	0.64 (0.33-1.26)	
*P* trend		.71		.51		.69		.96		.71		.44	
Triclosan													.61
≤5.27	210/410	1.00	22/28	1.00	72/166	1.00	24/40	1.00	53/56	1.00	39/120	1.00	
>5.27-≤20.57	160/369	0.87 (0.66-1.14)	7/11	0.52 (0.14-1.98)	73/196	0.86 (0.56-1.32)	18/15	1.84 (0.73-4.65)	35/61	0.51 (0.27-0.97)*	27/86	0.91 (0.49-1.71)	
>20.57	148/427	0.80 (0.60-1.06)	7/14	0.70 (0.20-2.54)	72/249	0.74 (0.49-1.13)	19/28	1.47 (0.63-3.43)	32/49	0.60 (0.31-1.16)	18/87	0.70 (0.35-1.40)	
*P* trend		.99		.51		.67		.84		.41		.26	
ΣMEP parabens													.79
≤47.51	230/395	1.00	12/13	1.00	84/176	1.00	21/17	1.00	65/84	1.00	48/105	1.00	
>47.51-≤195.19	160/397	0.82 (0.62-1.07)	10/20	0.80 (0.23-2.85)	67/204	0.82 (0.54-1.23)	27/26	0.82 (0.32-2.07)	34/48	0.92 (0.51-1.68)	22/99	0.81 (0.43-1.53)	
>195.19	128/414	0.73 (0.55-0.97)*	14/20	0.83 (0.25-2.78)	66/231	0.81 (0.54-1.22)	13/40	0.30 (0.11-0.79)*	21/34	0.76 (0.38-1.53)	14/89	0.80 (0.37-1.69)	
*P* trend		.002**		.37		.06		.01*		.57		.28	
Total parabens													.77
≤49.26	229/396	1.00	12/13	1.00	84/179	1.00	21/16	1.00	64/83	1.00	48/105	1.00	
>49.26-≤201.53	163/396	0.84 (0.64-1.10)	10/21	0.79 (0.22-2.77)	68/200	0.85 (0.57-1.28)	27/27	0.77 (0.30-1.97)	36/48	0.95 (0.53-1.73)	22/100	0.81 (0.43-1.54)	
>201.53	126/414	0.72 (0.54-0.96)*	14/19	0.85 (0.25-2.85)	65/232	0.80 (0.53-1.21)	13/40	0.29 (0.11-0.77)*	20/35	0.70 (0.35-1.42)	14/88	0.84 (0.39-1.80)	
*P* trend		.002**		.36		.07		.01*		.53		.29	
MEHP%													.59
≤5.49%	187/360	1.00	11/13	1.00	71/174	1.00	25/26	1.00	44/52	1.00	36/95	1.00	
>5.49-11.08%	181/407	0.93 (0.71-1.23)	16/17	0.77 (0.21-2.83)	78/198	1.03 (0.68-1.55)	19/29	0.72 (0.31-1.69)	43/61	1.03 (0.55-1.92)	25/102	0.79 (0.41-1.50)	
>11.08%	150/439	0.75 (0.56-0.99)*	9/23	0.30 (0.08-1.19)	68/239	0.74 (0.49-1.12)	17/28	1.29 (0.50-3.31)	33/53	0.85 (0.44-1.63)	23/96	0.78 (0.40-1.52)	
*P* trend		.09		.06		.22		.62		.42		.77	
MEHP/(MEOHP + MEHHP)													.62
≤9.76%	176/359	1.00	12/14	1.00	64/170	1.00	26/24	1.00	42/52	1.00		1.00	
>9.76-21.36%	186/410	1.01 (0.77-1.34)	16/17	1.00 (0.28-3.64)	77/202	1.06 (0.69-1.62)	21/30	0.68 (0.29-1.56)	44/57	1.08 (0.58-2.01)		1.16 (0.60-2.25)	
>21.36%	156/437	0.82 (0.62-1.08)	8/22	0.22 (0.05-0.94)*	76/239	0.89 (0.58-1.35)	14/29	0.84 (0.32-2.21)	34/57	0.85 (0.45-1.64)		1.04 (0.53-2.04)	
*P* trend		.18		.04*		.56		.56		.21		.99	
MEHP/(MECPP + MEHHP)													.79
≤7.87%	197/360	1.00	11/15	1.00	75/174	1.00	25/27	1.00	49/54	1.00	37/90	1.00	
>7.87-16.86%	174/412	0.91 (0.69-1.20)	16/17	0.82 (0.23-2.94)	75/199	0.99 (0.65-1.49)	21/28	0.85 (0.37-1.95)	37/58	0.98 (0.52-1.84)	25/110	0.70 (0.37-1.34)	
>16.86%	147/434	0.73 (0.55-0.97)*	9/21	0.37 (0.10-1.45)	67/238	0.72 (0.47-1.09)	15/28	1.13 (0.44-2.94)	34/54	0.84 (0.44-1.58)	22/93	0.73 (0.38-1.44)	
*P* trend		.05		.04*		.15		.59		.36		.70	
Phthalic acid													.38
≤36.90	159/386	1.00	8/9	1.00	82/239	1.00	12/19	1.00	39/46	1.00	18/73	1.00	
>36.90-≤79.76	174/405	0.94 (0.71-1.25)	9/23	0.19 (0.04-0.97)*	74/195	1.05 (0.71-1.57)	26/22	1.50 (0.54-4.15)	34/61	0.71 (0.36-1.37)	31/104	1.02 (0.49-2.12)	
>79.76	185/415	0.92 (0.70-1.23)	19/21	0.44 (0.10-2.01)	61/177	0.95 (0.63-1.44)	23/42	0.68 (0.25-1.83)	47/59	0.75 (0.39-1.42)	35/116	1.25 (0.61-2.56)	
*P* trend		.42		.77		.82		.15		0.97		0.53	
ΣDEHP													.39
≤63.21	171/404	1.00	18/25	1.00	70/211	1.00	16/23	1.00	44/58	1.00	23/87	1.00	
>63.21-≤133.05	194/399	1.14 (0.87-1.50)	12/16	0.78 (0.25-2.44)	77/209	1.09 (0.73-1.64)	25/20	1.63 (0.62-4.29)	45/57	1.10 (0.59-2.04)	35/97	1.25 (0.64-2.44)	
>133.05	153/403	0.89 (0.67-1.18)	6/12	0.68 (0.18-2.60)	70/191	1.11 (0.73-1.68)	20/40	0.45 (0.17-1.14)	31/51	0.77 (0.40-1.49)	26/109	0.96 (0.48-1.93)	
*P* trend		.59		.67		.58		.06		.89		.36	
ΣHMWP													.13
≤78.13	176/403	1.00	16/25	1.00	70/208	1.00	16/27	1.00	45/60	1.00	29/83	1.00	
>78.13-≤149.24	188/387	1.11 (0.84-1.46)	14/15	0.97 (0.31-3.01)	71/200	1.07 (0.71-1.62)	29/17	1.72 (0.66-4.52)	47/56	1.32 (0.72-2.45)	27/99	0.77 (0.40-1.51)	
>149.24	154/416	0.85 (0.64-1.13)	6/13	0.65 (0.17-2.43)	76/203	1.12 (0.74-1.68)	16/39	0.37 (0.14-0.97)*	28/50	0.77 (0.40-1.48)	28/111	0.74 (0.38-1.43)	
*P* trend		.42		.48		.72		.06		.81		.21	
ΣLMWP													.72
≤64.00	163/384	1.00	7/5	1.00	82/230	1.00	6/11	1.00	41/63	1.00	27/75	1.00	
>64.00-≤145.29	187/397	1.08 (0.81-1.42)	14/14	0.42 (0.08-2.20)	82/201	1.08 (0.73-1.60)	21/24	2.28 (0.66-7.92)	42/54	1.22 (0.66-2.27)	28/104	0.88 (0.45-1.73)	
>145.29	168/425	0.86 (0.64-1.15)	15/34	0.19 (0.04-0.93)*	53/180	0.86 (0.56-1.32)	34/48	1.60 (0.50-5.19)	37/49	0.91 (0.48-1.76)	29/114	0.92 (0.47-1.79)	
*P* trend		.06		.04*		.21		.45		.93		.40	
ΣLMHMPA													.57
≤226.33	175/379	1.00	10/11	1.00	82/219	1.00	12/13	1.00	48/60	1.00	23/76	1.00	
>226.33-≤442.34	173/397	0.98 (0.74-1.29)	11/20	0.31 (0.07-1.29)	74/194	1.17 (0.79-1.75)	24/24	0.76 (0.25-2.30)	33/54	0.72 (0.38-1.38)	31/105	1.08 (0.55-2.12)	
>442.34	170/430	0.78 (0.59-1.04)	15/22	0.41 (0.10-1.62)	61/198	0.84 (0.56-1.28)	25/46	0.37 (0.13-1.09)	39/52	0.71 (0.37-1.35)	30/112	1.05 (0.53-2.07)	
*P* trend		.06		.19		.45		.08		.68		.26	

Adjusted for breast cancer status, age (<65, ≥ 65–74, ≥ 75 years), BMI at urine collection (<25, ≥ 25–30, ≥ 30 kg/m^2^), and race and ethnicity (except in analyses stratified by race and ethnicity). All EDCs in units of ng/mg creatinine.

Abbreviations: BMI, body mass index; DEHP, di-2-ethylhexyl phthalate; het, heterogeneity; HMWP, high molecular weight phthalates; kg, kilograms; LMWP, low molecular weight phthalates; LMHMPA, low and high molecular weight phthalates and phthalic acid; m, meters; MECPP, mono(2-ethyl-5-carboxypentyl) phthalate; MEHHP, mono(2-ethyl-5-hydroxyhexyl) phthalate; MEHP, mono-2-ethylhexyl-phthalate; MEOHP, mono(2-ethyl-5-oxohexyl) phthalate; MEP, methyl-, ethyl-, and propylparabens; MetS, metabolic syndrome; OR, odds ratio.

^*^ *P* < .05 before Bonferroni corrections for multiple testing.

^**^ *P* < .003 statistically significant after Bonferroni corrections for multiple testing.

For phthalates, an inverse association with MetS was suggested for the highest tertile of MEHP% (>11.08% vs ≤5.49%: odds ratio [OR] 0.75; 95% CI 0.56-0.99, *P*_trend_ = 0.09, Table 2) and MEHP/(MECPP + MEHHP) (>16.86% vs ≤7.87%: OR 0.73; 95% CI 0.55-0.97, *P*_trend_ = .05) compared with the lowest tertile. Inverse associations for MEHP/(MEOHP + MEHHP) and MEHP/(MECPP + MEHHP) with MetS were suggested among African American women (*P*_trend_ = .04 for each) but were not statistically significant. No heterogeneity in the associations of phthalates and MetS was detected by race and ethnicity (*P*_heterogeneity_ > .13 for each).

Inverse associations with MetS were suggested for ΣMEP and total parabens among women with a normal/underweight BMI (*P*_trend_ = .01 for each, Table 3) and among women with an overweight BMI (*P*_trend_ = .01 for each), but the findings were not statistically significant. There were no statistically significant differences in the associations of ΣMEP or total paraben with MetS by BMI (*P*_heterogeneity_ = .33 and .44, respectively). We did not observe any statistically significant associations of phthalate metabolites and MetS by BMI or any significant heterogeneity in the associations of EDCs with MetS by BMI.

**Table 3.** Multivariable-adjusted odds ratios and 95% CIs for the association of urine BPA, triclosan, paraben, and phthalate metabolite measures with metabolic syndrome by body mass index among women in the multiethnic cohort study

	BMI at urine collection <25 n = 788		BMI at urine collection ≥25-<30 n = 572		BMI at urine collection ≥30 n = 368		*P*_het_
	MetS/No MetS	OR (95% CI)	MetS/No MetS	OR (95% CI)	MetS/No MetS	OR (95% CI)	
Bisphenol A							.29
≤0.84	29/239	1.00	79/137	1.00	92/50	1.00	
>0.84-≤1.76	31/223	1.18 (0.68-2.05)	68/114	1.08 (0.71-1.63)	59/51	0.62 (0.37-1.04)	
>1.76	37/227	1.40 (0.83-2.39)	59/113	0.96 (0.62-1.48)	64/52	0.68 (0.41-1.13)	
*P* trend		.18		.27		.46	
Triclosan							.50
≤5.27	35/218	1.00	83/125	1.00	92/67	1.00	
>5.27-≤20.57	27/207	0.78 (0.45-1.35)	58/118	0.67 (0.43-1.03)	75/44	1.21 (0.74-2.00)	
>20.57	35/264	0.79 (0.47-1.33)	65/121	0.73 (0.48-1.11)	48/42	0.83 (0.49-1.40)	
*P* trend		.96		.40		.35	
ΣMEP parabens							.33
≤47.51	34/186	1.00	86/130	1.00	110/79	1.00	
>47.51-≤195.19	34/221	0.92 (0.55-1.56)	69/129	0.79 (0.52-1.19)	57/47	0.82 (0.50-1.35)	
>195.19	29/282	0.57 (0.33-0.98)*	51/105	0.69 (0.44-1.08)	48/27	1.08 (0.60-1.95)	
*P* trend		.04*		.01*		.63	
Total parabens							.44
≤49.26	34/187	1.00	85/130	1.00	110/79	1.00	
>49.26-≤201.53	33/221	0.92 (0.54-1.55)	72/128	0.85 (0.56-1.28)	58/47	0.83 (0.50-1.37)	
>201.53	30/281	0.60 (0.35-1.03)	49/106	0.66 (0.42-1.04)	47/27	1.06 (0.59-1.90)	
*P* trend		.04*		.01*		.66	
MEHP%							.56
≤5.49%	31/194	1.00	72/107	1.00	84/59	1.00	
>5.49–11.08%	36/227	0.98 (0.58-1.66)	69/135	0.80 (0.52-1.22)	76/45	1.10 (0.66-1.84)	
>11.08%	30/268	0.65 (0.38-1.12)	65/122	0.81 (0.52-1.25)	55/49	0.70 (0.41-1.19)	
*P* trend		.75		.20		.12	
MEHP/(MEOHP + MEHHP)							.43
≤9.76%	27/194	1.00	72/106	1.00	77/59	1.00	
>9.76-21.36%	36/233	1.16 (0.67-1.99)	68/131	0.80 (0.52-1.23)	82/46	1.31 (0.79-2.18)	
>21.36%	34/262	0.86 (0.50-1.48)	66/127	0.77 (0.50-1.19)	56/48	0.80 (0.47-1.37)	
*P* trend		.81		.32		.11	
MEHP/(MECPP + MEHHP)							.60
≤7.87%	33/185	1.00	74/111	1.00	90/64	1.00	
>7.87-16.86%	35/234	0.83 (0.50-1.40)	70/135	0.82 (0.54-1.26)	69/43	1.08 (0.64-1.80)	
>16.86%	29/270	0.55 (0.32-0.94)*	62/118	0.78 (0.51-1.21)	56/46	0.78 (0.46-1.32)	
*P* trend		0.45		0.21		0.12	
Phthalic acid							.60
≤36.90	37/244	1.00	64/105	1.00	58/37	1.00	
>36.90-≤79.76	36/218	1.16 (0.71-1.92)	66/128	0.90 (0.58-1.40)	72/59	0.84 (0.48-1.46)	
>79.76	24/227	0.70 (0.40-1.21)	76/131	0.98 (0.63-1.52)	85/57	1.01 (0.58-1.74)	
*P* trend		.39		.70		.94	
ΣDEHP							.29
≤63.21	33/234	1.00	65/119	1.00	73/51	1.00	
>63.21-≤133.05	39/219	1.23 (0.74-2.04)	73/136	0.98 (0.64-1.50)	82/44	1.28 (0.76-2.17)	
>133.05	25/236	0.80 (0.46-1.40)	68/109	1.06 (0.68-1.64)	60/58	0.74 (0.44-1.26)	
*P* trend		.19		.51		.70	
ΣHMWP							.56
≤78.13	34/232	1.00	67/120	1.00	75/51	1.00	
>78.13-≤149.24	37/215	1.16 (0.70-1.94)	71/128	1.01 (0.66-1.55)	80/44	1.18 (0.70-2.00)	
>149.24	26/242	0.78 (0.45-1.35)	68/116	1.01 (0.66-1.55)	60/58	0.71 (0.42-1.20)	
*P* trend		.19		.79		.64	
ΣLMWP							.92
≤64.00	36/221	1.00	68/121	1.00	59/42	1.00	
>64.00-≤145.29	36/228	1.02 (0.61-1.69)	71/114	1.15 (0.74-1.76)	80/55	1.01 (0.59-1.72)	
>145.29	25/240	0.72 (0.41-1.26)	67/129	0.91 (0.58-1.41)	76/56	0.95 (0.55-1.65)	
*P* trend		.08		.41		.57	
ΣLMHMPA							.25
≤226.33	38/217	1.00	66/121	1.00	71/41	1.00	
>226.33-≤442.34	39/230	1.02 (0.62-1.68)	70/115	1.09 (0.71-1.68)	64/52	0.73 (0.42-1.25)	
>442.34	20/242	0.51 (0.29-0.92)*	70/128	0.97 (0.63-1.49)	80/60	0.78 (0.46-1.32)	
*P* trend		.046*		.54		.61	

Adjusted for breast cancer status, age (<65, ≥ 65–74, ≥ 75 years), and race and ethnicity. All EDCs in units of ng/mg creatinine.

Abbreviations: BMI, body mass index; DEHP, di-2-ethylhexyl phthalate; het, heterogeneity; HMWP, high molecular weight phthalates; LMWP, low molecular weight phthalates; LMHMPA: low and high molecular weight phthalates and phthalic acid; MECPP, mono(2-ethyl-5-carboxypentyl) phthalate; MEHHP, mono(2-ethyl-5-hydroxyhexyl) phthalate; MEHP, mono-2-ethylhexyl-phthalate; MEOHP, mono(2-ethyl-5-oxohexyl) phthalate; MEP, methyl-, ethyl-, and propylparabens; MetS, metabolic syndrome; OR, odds ratio.

^*^ *P* < .05 before Bonferroni corrections for multiple testing.

## Discussion

In this cross-sectional study of older, mostly postmenopausal women in the MEC, we observed an inverse association for ΣMEP and total parabens with MetS. There were no statistically significant differences in the association between any EDCs and MetS by race or BMI, and inverse associations with parabens (ΣMEP, total parabens) were suggested among women with normal and overweight BMIs.

Our null findings for BPA and triclosan are similar to another study among a population of women, the Early Life Exposure in Mexico to ENvironmental Toxicants (ELEMENT) Study, which reported no significant association between MetS and BPA, triclosan, or phthalate exposure [17]. But unlike our study, the ELEMENT study did not observe statistically significant associations between paraben measures and MetS [17]. The conflicting findings with the ELEMENT study may be explained, in part, by the age of study participants as the mean (SD) age of women in the ELEMENT study was 46.6 (6.3) years and 66.3 (7.9) years in our study. Differences in associations between MetS and EDCs by age and menopausal status have been reported. The National Health and Nutrition Examination Survey (NHANES) study (2001-2010) reported a statistically significant increased risk of MetS with exposure to ΣDEHP among women <50 years of age, but no association among women ≥50 years [16]. Differences by age have also been observed for EDCs and the components of MetS. In the Nurses’ Health Study, the association between BPA and type 2 diabetes differed by menopausal status (*P*_interaction_ = .03), displaying a positive association among premenopausal women (*P*_trend_ = .01) but not among postmenopausal women [6]. The association between EDCs and risk of MetS among older women remains unclear and additional studies of the mechanisms of paraben involvement in adipogenesis among older women are warranted to confirm the inverse association we observed with parabens and MetS among our mostly older, postmenopausal study population.

While some studies have found positive associations between individual classes of EDCs and MetS [15, 22], these studies were conducted in populations of men and women. In agreement with our findings, some prior studies among women have reported inverse associations of parabens with metabolic syndrome [9] and conditions that signify metabolic disorder such as type 2 diabetes [11] and BMI [9]. Parabens can accumulate in adipocytes and bind to estrogen receptors, therefore stimulating estrogenic activation and disrupting metabolic processes [31]. Parabens also dysregulate adipocyte differentiation via activation of the glucocorticoid receptor and peroxisome proliferator–activated receptor gamma [32]. Assessments of EDC exposures and adiposity, including body fat distribution, may aid in the understanding of the mechanisms by which parabens are associated with MetS.

Body fat distribution is an important anthropometric component of metabolic risk. With older age and menopausal onset, women often experience reduced circulating estrogen levels and a greater accumulation of visceral fat, which is associated with adverse metabolic outcomes [33, 34]. High EDC exposure also contributes to low testosterone levels, low sex hormone–binding globulin levels [20, 35], and increased insulin resistance [36], which may contribute to unfavorable body fat distribution profiles and increased adipogenesis that increases the risk of MetS [37, 38]. Since overall body size and fat distribution differ by demographic characteristics such as sex, age, and race and ethnicity [39], future studies should assess how differences in body fat distribution and visceral adiposity, contributes to differences in the association between EDCs and MetS by these factors.

The association between urinary phthalate measures and MetS was evaluated by race and ethnicity among non-Hispanic White, African American, and Mexican/Hispanic participants in the NHANES 2005-2014 population [18]. An inverse association between MEHP and MetS was observed among non-Hispanic White women [18]. We observed inverse associations between the MEHP ratios and MetS among African American women that were not statistically significant. African American women in the MEC had MEHP ratio measures that were among the highest by race and ethnicity (eg, mean MEHP/(MECPP + MEHHP) ratio measures in African American women were 11.8 ng/mg creatinine (95% CI 9.0-15.7) compared with 10.0 ng/mg creatinine (95% CI 7.8-12.8) in Latino women who had the lowest measures). Other NHANES studies have described high phthalate and paraben measures among African American women compared to non-Hispanic White women [40], and racial and ethnic differences in phthalate measures associated with personal care product use [41, 42]. Therefore, additional prospective analyses in multiethnic populations that assess potential sources of EDC exposure and the patterns of use of EDC products are needed to further explain racial and ethnic differences in EDC-MetS associations.

To our knowledge, this study is the first to evaluate the associations of secondary DEHP metabolite ratios and MetS among adult women. DEHP metabolites undergo hydroxylation and oxidation steps before excretion such that accounting for MEHP and secondary DEHP metabolites likely provides a more accurate indication of DEHP exposure [43, 44]. The inverse associations of parabens and MetS suggested among women with a normal/underweight BMI and women with an overweight BMI suggest possibly dampened effects of the EDCs on MetS in specific subgroups. Obesity was positively associated with MEHP measures and slowed the conversion of MEHP to the less toxic secondary phthalate metabolites in an NHANES study, but our finding of a suggestive inverse association among women of BMI <30 kg/m^2^ warrants further evaluation.

This study adds to the existing literature by evaluating multiple classes of EDCs in a multiracial and multiethnic population. We used comprehensive methods to quantify metabolite concentrations and we analyzed phthalate metabolite ratios, which have been understudied. We applied a conservative approach to estimating the associations by using a Bonferroni correction for all EDCs and detected some notable associations. A limitation of this study is the small samples sizes in analyses by race and ethnicity which may have contributed to increased uncertainty in the estimated effect sizes. We used a single urine sample for this assessment which may not be representative of long-term EDC exposure [45]. Also, urine collection methods varied slightly by study site; all samples collected in Los Angeles County were first morning samples whereas most samples from Hawai‘i were overnight collections. As there are potential additive effects of coexposure to multiple chemicals of similar toxicology, the Environmental Protection Agency is developing guidance for cumulative risk assessments for phthalates [46, 47]. This analysis may be improved by implementing the recommended methods once they are finalized and released. Also, we cannot eliminate the possibility that the findings of this cross-sectional study may be due to reverse causation.

Results from this cross-sectional assessment of the association between EDCs and MetS suggest an inverse relationship between total parabens and MetS among older women. This analysis adds to the growing literature that assesses specific detrimental health effects of EDCs and future studies should prospectively investigate the associations between EDCs and MetS among multiracial and multiethnic populations.

## Data Availability

Some or all datasets generated during and/or analyzed during the current study are not publicly available but are available from the corresponding author on reasonable request.

## Abbreviations

ΣDEHP: sum of all major DEHP metabolites
ΣHMWP: sum of high molecular weight phthalates
ΣLMHMPA: sum of all 10 phthalate metabolites and phthalic acid
ΣLMWP: sum of low molecular weight phthalates
ΣMEP: sum of methyl-, ethyl- and propylparaben
BMI: body mass index
BPA: bisphenol A
EDC: endocrine disrupting chemical
ELEMENT: ENvironmental Toxicants Study
DEHP: di-2-ethylhexyl phthalate
HDL: high-density lipoprotein
LLOD: lower limit of detection
MBP: mono-n-butyl phthalate
MBzP: mono-benzyl phthalate
MCHP: mono 2-carboxy-hexyl phthalate
MEC: Multiethnic Cohort Study
MECPP: mono(2-ethyl-5-carboxypentyl) phthalate
MEHHP: mono(2-ethyl-5-hydroxyhexyl) phthalate
MEHP: mono-2-ethylhexyl-phthalate
MEOHP: mono(2-ethyl-5-oxohexyl) phthalate
MEP: mono-ethyl phthalate
MetS: metabolic syndrome
MiBP: mono-isobutyl phthalate
MMP: mono-methyl phthalate
NHANES: National Health and Nutrition Examination Survey Study
OR: odds ratio
